# Does Prior Stroke Predict Long-Term Recurrent Stroke After Percutaneous Coronary Intervention? Five-Year Results From a Large Cohort Study

**DOI:** 10.3389/fneur.2021.740136

**Published:** 2021-11-02

**Authors:** Jing-jing Xu, Si-da Jia, Pei Zhu, Lin Jiang, Ping Jiang, Ying Song, Xue-yan Zhao, Jian-xin Li, Jue Chen, Yue-jin Yang, Run-lin Gao, Shu-bin Qiao, Bo Xu, Jin-qing Yuan

**Affiliations:** Department of Cardiology, Fuwai Hospital and Cardiovascular Institute, National Center for Cardiovascular Diseases, Chinese Academy of Medical Sciences and Peking Union Medical College, Beijing, China

**Keywords:** prior stroke, coronary heart disease, PCI—percutaneous coronary intervention, predictor, long-term outcome

## Abstract

**Background:** We found a positive correlation between the prior stroke history and recurrent stroke in patients who underwent percutaneous coronary intervention (PCI) in our previous study, which indicated the close interaction of stroke and cardiovascular diseases. However, it is unclear whether prior stroke is still associated with worse prognosis at a longer follow-up period.

**Methods:** A total of 10,724 coronary heart disease (CHD) patients who received PCI from January to December 2013 were prospectively enrolled and were subsequently divided into the prior stroke (*n* = 1,150) and non-prior stroke (*n* = 9,574) groups according to their history. Baseline characteristics and 5-year outcomes were recorded.

**Results:** Patients with prior stroke had more clinical risk factors, as well as more extensive coronary artery lesions. Although in-hospital outcomes were similar between patients from the two groups, the 5-year follow-up result revealed that patients with prior stroke experienced higher incidence of stroke, major adverse cardiac and cerebrovascular events (MACCEs), all-cause death, and cardiac death (7.0 vs. 3.0%, *p* < 0.001; 25.9 vs. 20.3%, *p* < 0.001; 5.3 vs. 3.5%, *p* = 0.002; 3.1 vs. 2.1%, *p* = 0.032, respectively). After the propensity score matching, the 5-year stroke rate was still higher in the prior stroke group (6.8 vs. 3.4%, *p* = 0.001). The multivariable regression analysis also identified the prior stroke as a risk predictor of the 5-year stroke (HR = 2.011, 95% CI: 1.322–3.059, *p* = 0.001).

**Conclusions:** Coronary heart disease patients with prior stroke who received PCI had a higher incidence of 5-year long-term adverse cardiovascular and cerebrovascular events, especially recurrent stroke. Prior stroke was a strong risk predictor of future stroke events.

## Introduction

The incidence of cerebrovascular diseases, especially stroke, has been increasing in low- and middle-income countries. Stroke is the leading cause of death and disability globally (1). Individuals with atherosclerosis have an increased risk of stroke (2). About 19% of stroke occurs simultaneously with cardiac events, and this, combined with cerebrovascular disease, may lead to worse prognosis for coronary heart disease (CHD) patients (3). As an essential means of revascularization, percutaneous coronary intervention (PCI) can improve therapeutic outcomes and the quality of life (4). However, the risk of recurrent cardio-cerebrovascular events increases in PCI patients with prior stroke, which complicates the decision on the treatment strategy choice (5, 6). As pointed out by Doehner et al. from the European Society of Cardiology Council on Stroke, the stroke care is an interdisciplinary challenge, and the close collaboration of stroke physicians and cardiologists is essential.

Our previous study reported that in CHD patients with prior stroke WHO received PCI, the incidence of 2-year major adverse cardiac and cerebrovascular event (MACCE), all-cause death, and other adverse events was higher than those without prior stroke, especially recurrent stroke, which remained at significantly higher risk after the confounder adjustment (7). However, with the extension of the period, whether the prior stroke is still related to the worse prognosis is not very unclear. Therefore, we further investigated the in-hospital outcomes and conducted a 5-year long-term follow-up to evaluate the exact impact of prior stroke on the prognosis of CHD patients after PCI, and we added subgroup analyses with high risk of clinical adverse events including older age, premature CHD (PCHD), and acute coronary syndrome (ACS) to assist the choice of clinical treatment regimens.

## Methods

### Study Population

A total of 10,724 consecutive CHD patients who received PCI from January to December 2013 were enrolled in this single-center study (Fuwai Hospital, National Center for Cardiovascular Diseases, Beijing, China). Patients were divided into prior stroke group (*n* = 1,150) and non-prior stroke group (*n* = 9,574). All patients were provided with written informed consent before the intervention, and the study has been approved by the Ethics Committee of the Hospital (ethical application no. IRB2012-BG-006, approval no. 2013-449).

### Procedural and Medications

The strategy, stent, and drugs used during PCI were at the physician's discretion. If there was no contraindication of taking long-term aspirin or P2Y12 inhibitors, selective patients received oral aspirin (loading dose 300 mg) and clopidogrel (loading dose 300 mg) or ticagrelor (loading dose 180 mg) at least 24 h before the procedure. Acute coronary syndrome patients [ST segment elevation myocardial infarction (MI) or non-ST elevation-ACS] scheduled for PCI received the same dose of aspirin and ticagrelor or clopidogrel (loading dose 300 or 600 mg) as soon as possible. Heparin of 1,000 IE was received at the beginning of the procedure; the 60 IE heparin/kg body weight was also conducted if the PCI has to be executed. Whether to use glycoprotein IIb/IIIa inhibitors was subject to operator's judgment. After the procedure, aspirin was prescribed at the dose of 100 mg per day indefinitely; clopidogrel 75 mg per day or ticagrelor 90 mg twice per day was advised for at least 1 year after PCI.

### Patient Follow-Up

All patients were evaluated by clinic visit or by phone at the first-month, sixth-month, first-year, second-year, and fifth-year time points after procedure. All adverse events were evaluated by a group of independent clinical physicians and were carefully checked and confirmed.

### Endpoints and Definitions

The primary endpoint was defined as MACCEs, a composite of all cause death, MI, stent thrombosis, repeat revascularization, and stroke during the 5-year follow-up. Secondary endpoint consisted of cardiac death, bleeding, and components of the primary endpoint. Death that could not be attributed to a non-cardiac etiology was considered as cardiac death. Myocardial infarction was defined by the third universal definition of MI (8). Revascularization was defined as the repeated revascularization for ischemic symptoms and events driven by PCI or surgery of any vessel. Unplanned target vessel revascularization (TVR) was defined as repeat percutaneous intervention or surgical bypass of any segment of the target vessel for ischemic symptoms and events driven (9). Stent thrombosis was defined according to the Academic Research Consortium definition, including definite, probable, and possible categories in the analysis (9). Bleeding was quantified according to the Bleeding Academic Research Consortium (BARC) definition criteria, including types 2, 3, and 5 in the analysis (10). The SYNTAX score is summarized from SYNTAX trial (Synergy between PCI with TAXUS drug-eluting stent and Cardiac Surgery) to compare the MACCE after coronary artery bypass grafting (CABG) or PCI. Scoring was performed by professional coronary intervention technicians, which was based on the qualitative and quantitative characterization of coronary artery disease by including 11 angiographic variables that take into consideration lesion location and characteristics. The treatment scheme was finally selected by the interventional operators (11).

### Statistical Analyses

**Figure 1:**
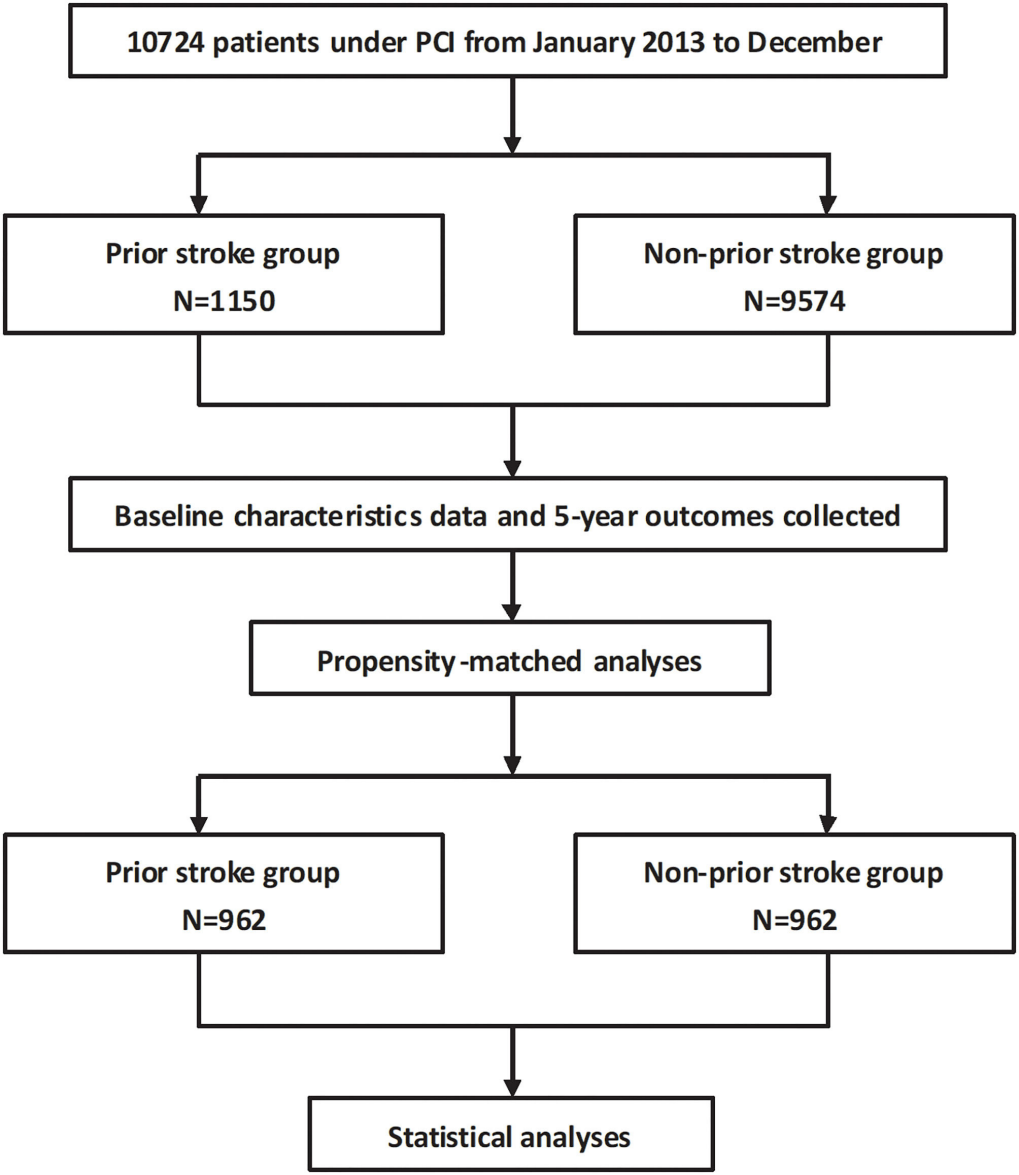
Flowchart.

Continuous variables were compared by Student's *t*-test as appropriate, and categorical variables were analyzed using Pearson's chi-square test. Continuous variables were presented as mean ± standard deviation, and categorical variables are presented as number with frequency. The 5-year survival curves were calculated using the Kaplan–Meier estimates and were tested using log-rank method for endpoints. Confounders were adjusted using the Cox proportional-hazards regression, where results were expressed as hazard ratios (HRs) with corresponding 95% confidence intervals (CIs). All clinically and statistically significant covariates were entered into the model with adjustments in the multivariate analysis. Due to substantial differences between the groups, we further performed a propensity score analysis that matched the following patient characteristics: age, gender, hypertension, diabetes mellitus, hyperlipidemia, smoking, prior MI, prior revascularization procedure, the SYNTAX score before PCI, and the residual SYNTAX score. Patients from the non-prior stroke group were matched with those from the prior stroke group according to their propensity score, resulting in 962 pairs of propensity-matched patients (*n* = 1,924 in total) in whom long-term outcomes were determined. Older age (≥75 years), PCHD (male ≤50 years, female ≤60 years), ACS, cardiac dysfunction [left ventricular ejection fraction (LVEF) ≤40%], hypertension, and diabetes mellitus were brought into subgroup analysis. The effect of the subgroup variables and prior stroke on the risk of long-term recurrent stroke was performed on Cox multivariate regression analysis. The results were expressed by the HR (95% CI) and interaction *p*-value. All statistical analyses were performed at a significance level of two-sided *p*-value = 0.05 with the software of SPSS version 19.0 (IBM Corporation, Armonk, New York). The flowchart is shown in Figure 1.

## Results

### Baseline Characteristics

A total of 10,724 CHD patients who have undergone PCI were enrolled in this study. The median follow-up time was 5.1 years (1,861 days) with a response rate of 91.5%. Among these patients, 1,150 (10.7%) had prior stroke, while 9,574 (89.3%) did not.

Baseline and angiographic characteristics of the two groups are displayed in Table 1. Compared with the non-prior stroke group, patients with prior stroke were older, contained more female, and were more likely to have CHD risk factors, such as hypertension, diabetes mellitus, and hyperlipidemia. They also had more extensive coronary artery disease (higher SYNTAX score before PCI and residual SYNTAX score), more prior MI, and revascularization history (Table 1). After propensity score matching, all baseline variables were comparable between the two groups, as shown in Table 1.

**Table 1:** Baseline and propensity score matched data of two groups.

	**Baseline**			**After propensity score matched analyses**		
	**Prior stroke group** ***n* = 1,150**	**Non-prior stroke group** ***n* = 9,574**	***P-*value**	**Prior stroke group** ***n* = 962**	**Non-prior stroke group** ***n* = 962**	***P-*value**
**Demographics**						
Age (years)	63.12 ± 8.90	57.80 ± 10.30	<0.001^*^	63.33 ± 8.73	63.43 ± 9.26	0.822
Female (%)	325 (28.3)	2,127 (22.2)	<0.001^*^	284 (29.5)	293 (30.5)	0.654
BMI (kg/m^2^)	25.98 ± 3.00	25.93 ± 3.20	0.57	25.94 ± 2.98	25.83 ± 3.16	0.449
**Risk factors**						
Hypertension (%)	941 (81.8)	5,965 (62.3)	<0.001^*^	786 (81.7)	795 (82.6)	0.592
Diabetes mellitus (%)	431 (37.5)	2,807 (29.3)	<0.001^*^	367 (38.1)	342 (35.6)	0.237
Hyperlipidemia (%)	854 (74.3)	6,357 (64.4)	<0.001^*^	713 (74.1)	704 (73.2)	0.641
Family history of CAD (%)	278 (24.2)	2,373 (24.8)	0.334	237 (24.6)	224 (23.3)	0.487
Smoker (%)	643 (56.4)	5,480 (58.7)	0.015	522 (54.3)	515 (53.5)	0.087
**Biochemical index**						
White blood cell (^*^109/L)	6.92 ± 1.94	6.92 ± 1.95	0.948	6.71 ± 1.70	6.69 ± 1.84	0.863
Hemoglobin (g/L)	138.10 ± 16.02	141.38 ± 15.84	<0.001^*^	138.48 ± 15.69	138.67 ± 16.03	0.799
Platelet (^*^109/L)	202.06 ± 54.04	203.74 ± 54.78	0.328	199.27 ± 51.08	198.11 ± 51.29	0.622
HbAlc (%)	6.74 ± 1.26	6.60 ± 1.24	0.001^*^	6.72 ± 1.19	6.76 ± 1.23	0.446
TC (mmol/L)	4.19 ± 1.05	4.21 ± 1.08	0.013^*^	4.10 ± 1.04	4.18 ± 1.07	0.088
LDL-C (mmol/L)	2.45 ± 0. 89	2.51 ± 0.91	0.024^*^	2.41 ± 0.87	2.47 ± 0.88	0.147
HDL-C (mmol/L)	1.03 ± 0.28	1.03 ± 0.28	0.824	1.04 ± 0.28	1.07 ± 0.29	0.240
TG (mmol/L)	1.69 ± 0.95	1.79 ± 1.10	0.004^*^	1.72 ± 0.99	1.73 ± 1.02	0.819
eGFR	80.05 ± 26.26	84.67 ± 28.26	<0.001^*^	79.89 ± 26.62	79.36 ± 28.23	0.673
**Diseased coronary vessels**						
Syntax score before PCI	12.25 ± 8.41	11.64 ± 8.08	0.016^*^	11.77 ± 8.01	11.96 ± 8.23	0.606
Residual SYNTAX score	4.19 ± 6.27	3.32 ± 5.61	<0.001^*^	4.02 ± 6.03	3.81 ± 5.95	0.431
LAD (%)	1,025 (89.1)	8,677 (90.6)	0.102	858 (89.2)	860 (89.4)	0.883
LCX (%)	227 (19.7)	1,606 (16.8)	0.012^*^	184 (19.1)	175 (18.2)	0.598
RCA (%)	220 (19.1)	1,689 (17.6)	0.212	181 (18.8)	178 (18.5)	0.861
Left main or three-vessel disease (%)	50 (4.4)	407 (4.3)	0.872	42(4.4)	45 (4.6)	0.809
**Cardiovascular conditions**						
ACS (%)	694 (60.3)	5,737 (59.9)	0.781	506 (52.6)	526 (54.7)	0.361
Cardiac shock (%)	7 (0.6)	23 (0.2)	0.025^*^	0 (0)	0 (0)	1.000
Previous MI (%)	251 (21.8)	1,810 (18.9)	0.010^*^	233 (24.2)	226 (23.5)	0.708
Previous PCI (%)	280 (24.3)	2,360 (24.7)	0.425	231 (23.7)	228 (24.0)	0.873
Previous CABG (%)	31 (2.7)	406 (4.2)	0.008^*^	30 (3.1)	26 (2.7)	0.587
LVEF (%)	62.57 ± 7.42	62.77 ± 7.35	0.425	63.546 ± 6.7988	63.414 ± 7.5874	0.690

*ACS, acute coronary syndrome; BMI, body mass index; CABG, coronary artery bypass grafting; eGFR, estimated Glomerular Filtration Rate; HBA1c, hemoglobin A1c; HDL-C, high density lipoprotein cholesterol; LAD, left anterior descending artery; LCX, left circumflex artery; LDL-C, low density lipoprotein cholesterol; LVEF, left ventricular ejection fraction; MI, myocardial infarction; PCI, percutaneous coronary intervention; SYNTAX score, synergy between percutaneous coronary intervention with taxus and cardiac surgery score; TC, total cholesterol; TG, triglyceride*.

*Data are expressed as mean ± standard deviation; or counts (percentage)*.

* *p < 0.05*.

### Outcomes

**Figure 2:**
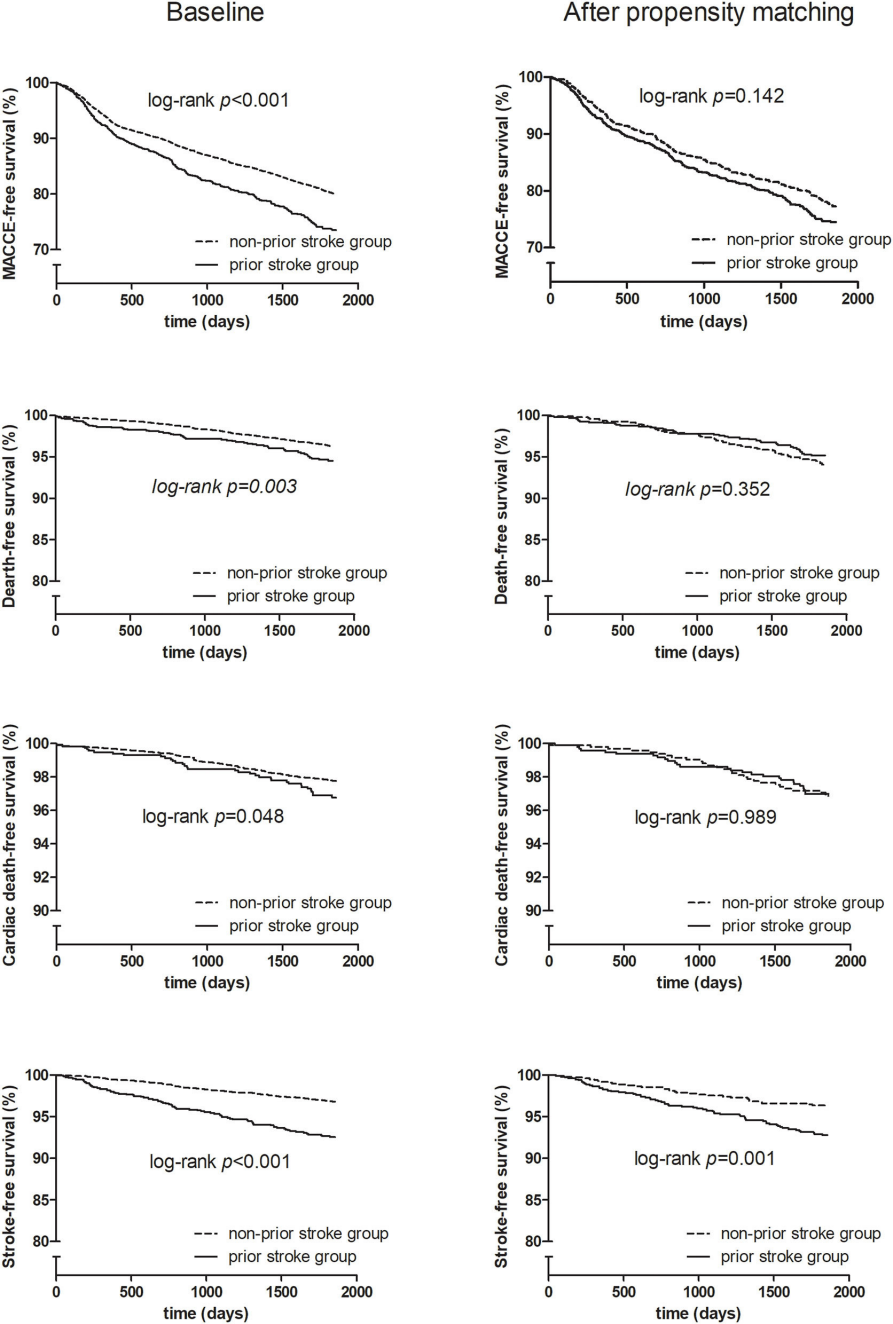
Kaplan–Meier survival curve for baseline and propensity score matching.

Before matching, unadjusted in-hospital and 5-year clinical outcomes are shown in Table 2. Patients with or without prior stroke had similar incidence of in-hospital adverse cardiovascular and cerebrovascular events. The 5-year follow-up showed significantly higher rates of MACCE, all-cause death, cardiac death, and stroke (25.9 vs. 20.3%, *p* < 0.001; 5.3 vs. 3.5%, *p* = 0.002; 3.1 vs. 2.1%, *p* = 0.032; 7.0 vs. 3.0%, *p* < 0.001, respectively) in the prior stroke group. After propensity score matching, however, there was no difference between two groups in the incidence of primary endpoint except for the 5-year stroke (6.8 vs. 3.4%, *p* = 0.001) (Table 2). The Kaplan–Meier estimates showed a similar trend (Figure 2).

**Table 2:** In-hospital and 5-year outcomes for baseline and propensity score-matched analyses.

	**In-hospital outcomes**						**5-Years outcomes**					
	**Baseline**			**After propensity score matched analyses**			**Baseline**			**After propensity score matched analyses**		
	**Prior stroke group ** ***n* = 1,150**	**Non-prior stroke group *n* = 9,574**	***p*-value**	**prior stroke group ** ***n* = 962**	**Non-prior stroke group *n* = 962**	***p*-value**	**prior stroke group ** ***n* = 1,150**	**Non-prior stroke group *n* = 9,574**	***p*-value**	**prior stroke group ** ***n* = 962**	**non-prior stroke group *n* = 962**	***p*-value**
MACCE	11 (1.0)	148 (1.5)	0.118	6 (0.6)	19 (2.0)	0.009^*^	298 (25.9)	1,940 (20.3)	<0.001^*^	240 (24.9)	222 (23.1)	0.337
All cause death	5 (0.4)	18 (0.2)	0.087	2 (0.2)	2 (0.2)	1.000	61 (5.3)	336 (3.5)	0.002^*^	45 (4.7)	54 (5.6)	0.353
Cardiac death	3 (0.3)	15 (0.2)	0.415	1 (0.1)	2 (0.2)	0.563	36 (3.1)	205 (2.1)	0.032^*^	28 (2.9)	28 (2.9)	1.000
Recurrent MI	6 (0.5)	130 (1.4)	0.017^*^	4 (0.4)	17 (1.8)	0.004^*^	60 (5.2)	511 (5.3)	0.864	51 (5.3)	56 (5.8)	0.619
Stent thrombosis	3 (0.3)	19 (0.2)	0.658	2 (0.2)	3 (0.3)	0.654	–	–	–	–	–	–
TVR	–	–	–	–	–	–	155 (13.5)	1,265 (13.2)	0.802	131 (13.6)	132 (13.7)	0.947
Stroke	1 (0.1)	2 (0.1)	0.206	0 (0)	0 (0)	1.000	80 (7.0)	284 (3.0)	<0.001^*^	65 (6.8)	33 (3.4)	0.001^*^
Bleeding	1 (0.1)	11 (0.1)	0.789	0 (0)	2 (0.2)	0.157	159 (13.8)	1,286 (13.4)	0.712	138 (14.3)	129 (13.4)	0.553

*MACCE, major adverse cardiac and cerebrovascular events; MI, myocardial infarction; TVR, revascularization*.

* *p < 0.05*.

The multivariable regression analysis identified prior stroke as a risk predictor of 5-year stroke before the propensity score matching (HR = 1.962, 95% CI: 1.520–2.532, *p* < 0.001). Prior stroke remained a strong independent risk predictor of 5-year stroke after the propensity score matching (HR = 2.011, 95% CI: 1.322–3.059, *p* = 0.001) (Table 3).

**Table 3:** Multivariable regression analyses of prior stroke for 5-years long-term prognosis.

	**Baseline**			**After propensity score matched analyses**		
	**HR**	**95% CI**	***p*-value**	**HR**	**95% CI**	***p*-value**
MACCE	1.200	1.059–1.360	0.004^*^	1.093	0.911–1.312	0.339
All cause death	1.119	0.848–1.477	0.427	0.879	0.591–1.308	0.525
Cardiac death	1.079	0.753–1.547	0.679	1.076	0.636–1.820	0.785
Recurrent MI	0.919	0.700–1.207	0.543	0.898	0.614–1.312	0.578
TVR	1.062	0.896–1.260	0.489	0.994	0.780–1.266	0.958
Stroke	1.962	1.520–2.532	<0.001^*^	2.011	1.322–3.059	0.001^*^
Bleeding	1.061	0.896–1.255	0.493	1.080	0.849–1.374	0.529

*MACCE, major adverse cardiac and cerebrovascular events; MI, myocardial infarction; TVR, revascularization.*

* *p < 0.05*.

### Subgroup Analysis

**Figure 3:**
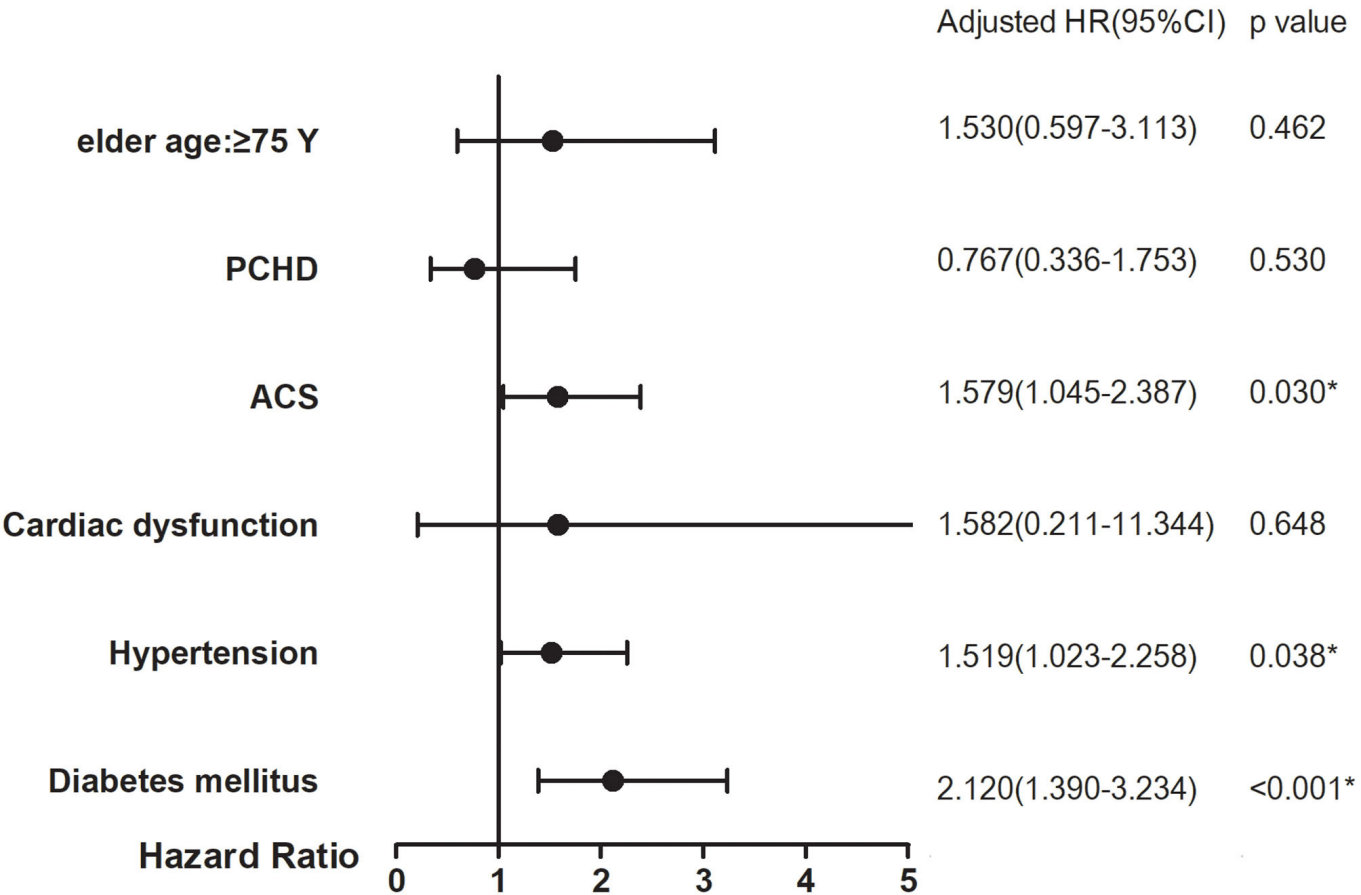
Subgroup analyses on 5-year stroke between prior stroke and non-prior stroke. *p*-Value for interaction in each subgroup analysis for adjusted HRs. *p < 0.05.

The subgroup analyses with high risk of clinical adverse events included those with age ≥75 years, PCHD, ACS, cardiac dysfunction, hypertension, and diabetes mellitus. They showed that ACS, hypertension, and diabetes mellitus had significant interaction with prior stroke history to increase the risk of 5-year stroke (HR = 1.579, 95% CI: 1.045–2.387, interaction *p* = 0.030; HR = 1.519, 95% CI: 1.023–2.258, interaction *p* = 0.038; HR = 2.120, 95% CI: 1.390–3.234, interaction *p* < 0.001, respectively) (Figure 3).

## Discussion

This is a study focusing on the prognosis of CHD patients with or without prior stroke who underwent PCI in the largest cardiovascular center in China. The major findings of this study were as follows: (1) it was common that CHD patients undergoing PCI had a prior stroke history; (2) patients with prior stroke had more high-risk baseline characteristics and were more likely to suffer severe coronary artery diseases; (3) although the incidence of in-hospital MACCE with or without prior stroke was similar, the 5-year follow-up showed a significantly higher incidence of recurrent stroke and MACCE in patients with prior stroke; (4) for CHD patients who underwent PCI, prior stroke was a strong risk predictor of future stroke events.

### Prevalence of Prior Stroke in Coronary Heart Disease Patients Undergoing Percutaneous Coronary Intervention

Prevalence of stroke varies in nations around the globe, where in China the rate is reported to be 2.6–7.2 per 1,000 persons (12). Stroke and CHD usually share many risk factors, and CHD patients are frequently complicated by stroke. Previous studies reported that the proportion of patients with CHD combined with prior stroke/transient ischemic attack (TIA) is about 13.3–19.7%, which is related to race, age, and other factors (13, 14). In our cohort of CHD patients undergoing PCI in the largest cardiovascular center in China, we found that 10.7% of the patients had a history of prior stroke, which reflected the real-world situation of China.

### High-Risk Clinical Characteristics of Patients With Prior Stroke

Patients with cardiovascular diseases and prior stroke were more likely to have more atherosclerotic risk factors, such as higher age, diabetes mellitus, hypertension, hyperlipidemia, chronic kidney disease, and peripheral arterial disease (15). Similarly, CHD patients with prior stroke in our study had worse baseline characteristics, including higher age, female gender, and more CHD risk factors such as hypertension, diabetes, hyperlipidemia, smoking, and much complex coronary anatomy. These factors are closely related to the occurrence and development of atherosclerosis and are associated with the increase in adverse cardiovascular and cerebrovascular events. The high-risk characteristics of patients with prior stroke undergoing PCI call for a better line of treatment strategy from the heart.

### Worse Long-Term Prognosis of Patients With Prior Stroke

Stroke has become the leading cause of death and disability-adjusted life-years lost in China (16). But for CHD patients with prior stroke who underwent PCI, the prognosis remained controversial. Some studies found that patients with prior stroke usually suffered from more events and higher mortality (17, 18). Others showed similar incidence of non-cerebrovascular adverse events between patients with or without prior stroke and that PCI was considered to be safe and effective for CHD patient with prior stroke (7, 19).

In this study, although in-hospital outcomes were similar between the two groups, the incidence of adverse cardiovascular and cerebrovascular events of patients with prior stroke was significantly higher during a long-term follow-up of 5 years. This might be due to more risk factors and more multi-vessel disease in patients with prior stroke. In our propensity-matched cohort, however, no difference in 5-year ischemic and hemorrhagic events was found between the two groups, with the exception of recurrent stroke. In general, patients with prior stroke were at a higher risk of cardio-cerebrovascular ischemic and hemorrhagic events, especially recurrent stroke.

### Prior Stroke as a Strong Risk Predictor of Future Stroke

Known for its high prevalence, mortality, and disability, stroke has also been infamous for high rate of recurrence (20). Prior stroke was considered to be a risk factor of recurrent stroke (21, 22). In the current study, the multivariable analysis identified prior stroke as an independent risk predictor of future stroke, even after propensity score matching. In addition, this study also shows a significant interaction between ACS, hypertension, diabetes, and prior stroke history, which will significantly increase the risk of future stroke. Hypertension and diabetes history are traditional risk factors of cardiovascular and cerebrovascular diseases. It is suggested that the risk of future stroke in patients combined with prior stroke history and cardiovascular and cerebrovascular risk factors will persist in the long-term follow-up.

### Potential Clinical Implications

This study suggested that it was typical for CHD patients undergoing PCI to have a prior stroke history. These patients had more risk factors of atherosclerosis and more complex vascular condition. Despite standard secondary prevention, patients with prior stroke fared worse in the long run, mainly due to an elevated risk of recurrent stroke. Even after confounder adjustment with both propensity matching and multivariable regression, risk of recurrent stroke in patients with prior stroke remained about two-fold higher than in those without stroke history. Therefore, it is reasonable to suggest that such patients need to receive PCI. A preprocedural consultation involving neurologists and interventional cardiologists might provide a better treatment regimen for additional long-term benefit.

## Limitation

Several limitations have to be taken into consideration in this study. First, the data of this study came from a single clinical center, which might not represent the general population. Second, the population of this study is mainly Asian patients, which may be a reason of an expected higher prevalence of intracranial atherosclerosis as a cause of stroke and an evident link with coronary atherosclerosis, but it is challenging to extend to other populations. Third, the prior stroke history was self-reported by patients, and we cannot confirm whether it is hemorrhagic or ischemic. Otherwise, the risk of recall bias would exist. Fourth, the observational design of this study with unmeasured confounders may preclude the definitive conclusion, and additional study may be needed to confirm the observations.

## Conclusion

With standard secondary prevention, CHD patients with prior stroke who underwent PCI still had a higher incidence of 5-year long-term adverse cardiovascular and cerebrovascular events, especially stroke. Prior stroke was a strong risk predictor of future stroke events.

## Data Availability Statement

The raw data supporting the conclusions of this article will be made available by the authors, without undue reservation.

## Ethics Statement

The studies involving human participants were reviewed and approved by Ethical Application No.: IRB2012-BG-006, Approval No.: 2013-449. The patients/participants provided their written informed consent to participate in this study.

## Author Contributions

J-jX: contributed to project design and article writing. S-dJ, PZ, LJ, PJ, and YS: contributed to data collection and arrangement. X-yZ, JC, and R-lG: contributed to project design. J-xL: contributed to data sorting and statistics. S-bQ and Y-jY: contributed to project design and operation. BX: project design and overall plan. All authors contributed to the article and approved the submitted version.

## Conflict of Interest

The authors declare that the research was conducted in the absence of any commercial or financial relationships that could be construed as a potential conflict of interest.

## Publisher's Note

All claims expressed in this article are solely those of the authors and do not necessarily represent those of their affiliated organizations, or those of the publisher, the editors and the reviewers. Any product that may be evaluated in this article, or claim that may be made by its manufacturer, is not guaranteed or endorsed by the publisher.
